# Mixed methods evaluation of well-being benefits derived from a heritage-in-health intervention with hospital patients

**DOI:** 10.1080/17533015.2013.800987

**Published:** 2013-05-17

**Authors:** Hannah L. Paddon, Linda J.M. Thomson, Usha Menon, Anne E. Lanceley, Helen J. Chatterjee

**Affiliations:** a University College London, UCL Museums & Public Engagement, Darwin Building, Gower Street, London, UK; b Department of Women's Cancer, UCL Elizabeth Garrett Anderson Institute for Women's Health, London, UK

**Keywords:** museum object handling, material objects, mixed methods, wellbeing, happiness

## Abstract

**Background:**

This study sought to determine the effects of a heritage-in-health intervention on well-being. Benefits of arts-in-health interventions are relatively well-documented yet little robust research has been conducted using heritage-in-health interventions, such as those involving museum objects.

**Methods:**

Hospital patients (*n* = 57) participated in semi-structured, 30–40 minute facilitated interview sessions, discussing and handling museum objects comprising selections of six artefacts and specimens loaned from archaeology, art, geology and natural history collections. Well-being measures (Positive Affect Negative Affect Scale, Visual Analogue Scales) evaluated the sessions while inductive and deductive thematic analysis investigated psycho-educational features accounting for changes.

**Results:**

Comparison of pre- and post-session quantitative measures showed significant increases in well-being and happiness. Qualitative investigation revealed thinking and meaning-making opportunities for participants engaged with objects.

**Conclusions:**

Heritage-in-health sessions enhanced positive mood and social interaction, endorsing the need for provision of well-being-related museum and gallery activities for socially excluded or vulnerable healthcare audiences.

## Background

Arts-in-health interventions have received substantial attention in recent years (Cox et al., 2010; Clift et al., 2009; Sonke, Rollins, Brandman & Graham-Pole, 2009; Staricoff, 2004, 2006; Wreford, 2010) and encompass a wide variety of cultural activities aiming to enhance individual and community welfare, healthcare delivery and healthcare environments. Arts Council England (2007, pp. 5–6), found a “considerable and growing evidence base of the effectiveness of arts interventions in healthcare and in promoting well-being” that included improving the “mental, emotional and spiritual state of Health Service users” and “help medical staff, caregivers, patients and families to communicate more effectively with each other by offering opportunities for social interaction, involvement and empowerment”. It is increasingly accepted that health, well-being and quality of life are reliant upon interconnections between physical, psychological and social functioning. Although this view is broadly in keeping with the World Health Organization (1948) definition; “health is a complete state of physical, mental and social well-being, not merely an absence of disease or infirmity”, the word “complete” can be debated in the light of an ageing population, better screening techniques and an increase in the diagnosis of chronic disease. The WHO definition underestimates the human capacity to adapt to physical, emotional and social change, and to experience well-being in the presence of disease or disability. The definition of well-being is even more ambiguous and controversial (Carlisle & Hanlon, 2007). The New Economics Foundation (a UK Government think-tank) define well-being as “the dynamic process that gives people a sense of how their lives are going, through the interaction between their circumstances, activities and psychological resources or ‘mental capital’” (NEF, 2009, p. 3). This definition is adopted here since museums and galleries appear to identify with NEF's view of well-being (Chatterjee & Noble, 2013) by endorsing NEF's five “actions” to improve well-being in everyday life: “be active”, “connect”, “keep learning”, “give” and “take notice” (NEF, 2008).

Heritage-in-health interventions offered by museums (including art galleries) exemplify the NEF view of well-being, particularly the actions concerned with connecting and learning seen to promote renewed confidence and enjoyment. Heritage-in-health interventions are similarly broad ranging as art-in-health interventions but involve a heritage element such as museum objects and artworks, historic buildings and heritage sites. However, comparatively few studies have been carried out to determine the effectiveness of heritage-in-health interventions in promoting well-being (Camic & Chatterjee, 2013). A study in which hospital patients were invited to explore objects from museum loan boxes with a facilitator at their bedsides showed increases in self-report measures of life satisfaction and health status (Chatterjee, Vreeland & Noble, 2009). Museum object handling sessions carried out with hospital patients by medical students showed improvements in patient quality-of-life measures and student communication, observation and research skills (Chatterjee & Noble, 2009; Noble & Chatterjee, 2008). A study of museum object handling with cancer patients using quantitative measures demonstrated significant improvements in patient psychological well-being and happiness (Thomson, Ander, Menon, Lancely & Chatterjee, 2012). Furthermore, qualitative research revealed that when experienced nurses took museum objects to patients’ bedsides, the objects acted as a vehicle for communication and emotional disclosure in women facing a gynaecological cancer diagnosis (Lanceley et al., 2012). These studies complement work associated with the role of art galleries in health and well-being, which has also received attention in recent years (Eekelaar, Camic & Springham, 2012; Roberts, Camic & Springham, 2011; Rosenberg, 2009).

## Theoretical Framework

The multi-disciplinary study reported here used a mixed-methods approach to assess the well-being benefits of handling and discussing museum objects with a range of hospital patients. The study sought to quantify the effects of a heritage-in-health intervention using clinically accepted scales derived from psychological and medical practice. It used qualitative thematic analysis of audio recordings to provide in-depth understanding of the processes involved in engaging with heritage objects and to see if and how this engagement could lift mood and enhance well-being. Theoretical bases drawn from arts-in-health, psychology and educational research provided a conceptual framework for the study. Simmons (2006, pp. 2–4) suggested that arts-in-health practices are grounded in two theoretical approaches, “dual coding” (Baddeley & Hitch, 1974; Paivio, 1971, 1986) and the “contiguity effect” (Clark & Paivio, 1991). Both theories were derived from psychological research into memory and are seen as reliant upon the interaction between sensory modalities. Within Paivio's (1971) concept of dual coding, verbal and visual material is connected in a short-term store or “working memory” during encoding and are subsequently integrated with material retrieved from long-term memory (Paivio, 1986). Within the contiguity effect, performance is enhanced when verbal and visual material is coordinated, not presented separately, a process attributed to the formation of better connections in the brain (Clark & Paivio, 1991).

Similarly, Baddeley (1986, 1992) hypothesized a two-component working memory store comprising auditory memory or “echoic store” and visual memory or “iconic store”. Each store has limited capacity; hence, an encoding strategy that draws simultaneously upon both stores should demonstrate a “modality effect” in reducing the cognitive load of one and exploiting available capacity in the other. As an alternative to the concept of memory stores, Craik and Lockhart (1972) advocated a “levels of processing” approach where “deeper” encoding of information leads to the formation of more connections in the brain than “shallower” encoding. Their two-stage model consisted of “maintenance rehearsal”, where material is retained only long enough to use it and “elaborative rehearsal”, where material is processed more deeply for subsequent retrieval from memory. Given that many arts-in-health interventions combine conversation with visual exploration, they may draw upon the modality effect in tapping into available capacity.

In addition to hearing and vision, heritage-in-health interventions involve senses such as touch and smell, theoretically implicating a model of multiple coding rather than dual coding. A multiple coding concept is relevant for older healthcare recipients with sensory decline (e.g. stroke, macular degeneration, etc.) because if one or more of the senses is compromised, it may be important to maximize communication and social contact during the session through other available cognitive channels. Spector, Orrell and Woods (2008) showed that twice-weekly cognitive stimulation therapy (CST) with older adults diagnosed with early stage dementia living in residential care led to increases in two measures of cognition (Mini Mental State Examination and Alzheimer's Disease Assessment Scale – Cognition) and in a participant-rated quality of life measure (Quality of Life Scale in Alzheimer's Disease), when compared with no treatment. CST employed sensory stimulation using a variety of stimuli such as social history objects and sound to prompt discussion and reminiscence. Its authors believe CST impacts upon cognitive processing and neuronal growth by tapping into multiple biological, psychological and social factors.

Educational research into stimulating and integrating sensory modalities, particularly the deployment of VAK (visual, aural or kinaesthetic/tactile) preferences associated with the Montessori Method, a method of educating children that stresses the development of initiative and natural ability (Kilpatrick, 1914) demonstrated wider appeal and assimilation of learning from the multiple presentation of material. Educational theory suggests learning is a cognitive process by which skill or knowledge is acquired associated with behavioral change and positive effects on mood (Uljens, 1997). Furthermore, Hein (1999) suggests that learners are active rather than passive in their acquisition of information. Constructivist theories, originating from the early twentieth century work of Piaget (Piaget, Eames & Brown, 1982) and Vygotsky (Vygotsky & Kozulin, 1986) suggest that children learn by building upon knowledge already acquired through their prior experience of the world (Pass, 2004). Although these educational theories are associated with childhood development and acquisition of knowledge, the more recent emphasis on lifelong learning has necessitated reference to constructivist frameworks and models of adult learning (e.g. Falk & Dierking, 2000; Hein, 1999) based on theories originally conceptualised for younger learners. Field (2009) showed that adult participation in lifelong learning had a direct effect on well-being by encouraging people to develop resources and cognitive capacities, an indirect impact where people could thrive and increase their resilience to risk, and a cumulative effect by influencing the social and economic environment. Within the area of learning skills, Brown and Wragg (1993) determined that the main reasons for asking questions were cognitive, affective and social, and dealing with knowledge, feelings and relationships.

The role of material objects in “meaning-making” is relevant to the study. Rowe (2002) attributes this to their ability to function as a “mental representation of possible relationships among things, events, and relationships. Humans bring their own knowledge, experiences and values to objects and make meaning” (Baumeister, 1991, p 15). Material objects also elicit a sense of identity and play a role in the development of self-awareness through multisensory interaction (Vygotsky, 1933 [2002]; see Camic, 2010; Romano, McCay & Boydell, 2012). The work of Camic, Brooker and Neal (2011) on “found objects” (referred to as material objects that are found or discovered, are not usually purchased, hold no intrinsic financial value and have personal significance) showed that the use of such objects in psychotherapy helped to enhance engagement, increase curiosity, reduce difficult feelings, evoke memories and provide a sense of agency through increased physical activity and environmental action. Furthermore, several authors have suggested that museum objects trigger memories, ideas and emotions in ways that other information-bearing materials do not (Chatterjee & Noble, 2013; Kavanagh, 2000; Lanceley et al., 2012; Phillips, 2008). Pearce (1995) argued that museum objects function as symbols of identity, relationships, nature, society and religion, and Dudley (2010) suggested that multisensory museum object encounters elicit ideas and meaning-making opportunities. Froggett et al. (2011) conducted an evaluation of health and well-being programmes run by several museums in the North West of England. The study determined that when individuals interact with museum objects the intrinsic, physical and material properties of the objects trigger sensory, emotional and cognitive associations, memories and projections. This is further exemplified by Newman and McLean (2006), Ander et al. (2012) and Lanceley et al. (2012) in studies focused on assessing the impact of museum object encounters on well-being.

The above psycho-educational theories acted as a conceptual framework in which the perception of well-being derived from the object handling sessions was assessed. The study examined quantitative and qualitative changes in psychological well-being resulting from handling and discussing museum objects in on-to-one, facilitated sessions. The research question asked if standardized psychological measures of well-being and happiness would be improved as a result of a museum object handling session and whether qualitative methods could be used to investigate the aspects of these sessions that led to the predicted benefits. The aim of the research was to describe typical features of this intervention, consider the factors that influenced the patients’ contributions to the sessions and examine the relationship of these factors to immediate, post-session, psychological well-being outcomes, in relation to the psycho-educational theories explored above.

## Methods

### Participants

The study was conducted with volunteer inpatients from a large, central London NHS Foundation Trust hospital over a 6-month period. Participants were of mixed gender, ethnicity and social background and spoke English sufficiently well to understand the patient information leaflet. All participants gave their informed consent to take part prior to inclusion in the study and for audio recording.

### Materials

The choice of quantitative measures was based upon a review of scales for evaluating psychological well-being, quality of life and perceived health status in healthcare settings (Thomson, Ander, Menon, Lanceley & Chatterjee, 2011). The review indicated that the most suitable self-report measures for assessing well-being at patients’ bedsides were the Positive Affect Negative Affect Scale (PANAS) (Watson, Clark & Tellegen, 1988), and the Visual Analogue Scale (VAS) (EuroQol Group, 1990). Six boxes were compiled that each contained six museum objects displayed in conservation materials. Objects comprised archaeological and ethnographic artefacts, etchings and printing plates, fossils, mineral samples and zoology specimens that varied in their tactile, visual and kinaesthetic properties (e.g. Egyptian bronze figurine, Neolithic hand axe, 1950s print, fossilized shark's tooth, piece of agate and turtle carapace).

### Design

A mixed-methods approach to data collection and analysis was used. The study examined changes in mood pre- and post-session using the PANAS to measure psychological well being (10 positive and 10 negative mood adjectives each rated from 1 to 5) and two VAS scales to assess subjective well-being and happiness (each estimated out of 100). Qualitative methods were used to investigate the processes that may lead to engagement with the objects and to well-being benefit.

Quantitative, multivariate analyses of variance were carried out on the PANAS and VAS scores from adult participants (*n* = 57) in four inpatient groups: Acute and Elderly Care (*n* = 11), General Oncology (*n* = 16), Gynaecological Oncology (*n* = 16) and Neurological Rehabilitation (*n* = 14), using the statistical software package SPSS (Statistical Package for the Social Sciences) 17.0 (2007).

Content and thematic analyses carried out on the recorded discourse from 16 sessions with participants, selected to represent the 4 inpatient groups and considered typical of the data overall, were entered into the qualitative analysis using the qualitative analysis software NVivo 8 (QSR International, 2008). Data were first subjected to content analysis to summarize the use of positive and negative mood adjectives during the object handling session (Krippendorff, 2004). The analysis was performed using the keyword search function in NVivo and involved examining the frequency with which PANAS adjectives, alternate forms of these words or synonyms occurred during the session.

A second-stage thematic analysis was used to bring out individual, personal ways in which patients engaged with the objects and how each session was facilitated. All transcripts were independently coded by one researcher (HP) and concerned particular responses and reactions. Codes were grouped into more detailed themes to understand the interaction more fully (Braun & Clark, 2006; Patton, 1990). Analysis was both inductive and deductive because the semi-structured format of the sessions ensured that predetermined areas were covered while allowing emergence of new concepts from the participants. A coding manual was produced in which the codes, their definitions and relationship to themes, with text examples, were documented (Table 1) in accordance with accepted analytic practice methods (Joffe, 2011). Two researchers (AL; HC) who were not involved in the sessions, tested the coding manual for validity and inter-rater reliability using the same transcript (Appendix 1) and discussed any differences. Agreement was high, but where minor discrepancies arose, discourse was reread and discussed until agreement was reached. There was agreement after scrutiny of 16 interactions that no new codes were emerging and that data analysis had reached saturation (Holloway & Wheeler, 2010).

**Table 1.** Coding manual.

Facilitator code	Explanation	Patient code	Explanation
Confirming patient thinking	This is very similar to agreeing. Facilitators confirm facts or simply agree with what patients are saying (this may be split into agreeing vs. confirming patient thinking as I think there may be grounds for doing so).	Being correct	Patients are keen to be correct about objects – if not, maybe this is construed by the patient as unfavourable or that they are stupid, silly, etc. It can make a difference to the amount of participation they have in a session and whether they enjoy it or not.
Questioning	Facilitators using questions to stimulate conversation based around the objects and also to find out more about the patient; their background and level of knowledge.	Questioning	Patients question the facilitators about the objects, ‘how old is it? Where does it come from?’ etc. They may also question facts that the facilitator gives them, sometimes rhetorically but in some instances because they doubt the information that has been given to them or want to know more about where the facilitator got the information from. “But how do you know?”
Giving information	Facilitator gives facts about objects and can often, quite literally, hand over object information sheets.	Giving information	Patient gives facts about his or her life.
Inviting touch	In order to get patients interacting and engaging with the objects, facilitators invite the patients to touch the objects. Often they encourage them to feel textures, gauge weight or understand its fragility.	Enjoying session	Patient may laugh or joke with facilitator. Indication that they are enjoying the session. They may also overtly acknowledge enjoyment.
Sharing power/passing over control	This is mainly seen when facilitators invite patients to select an object. By doing this at the start of the session in particular, it shows willingness from the facilitator to be led by the patient but also sets them on more equal ground. Patients are more likely to engage with things they select as they often remark “I'm interested in that one”.	Agreeing	Agreeing with the facilitator can be a simple acknowledgement of hearing to a firm acknowledgement followed by an observation, fact or question.
Hearing impaired	This is important in elderly and acute illness sessions. Hearing might be impaired because of medical problems, distractions in ward, quiet responses from facilitators.	Seeking validation	Patients who treat the object identification as a guessing game, and even those that aren't, seek validation that they are correct with guesses and facts. They look to the facilitator to confirm their thoughts. This code links to “confirming patient thinking”, “being correct” and “guessing game”. Facilitator as expert.
Selecting objects	Selected objects because of colour, size, shape (features) or they were curious about what it was. They also selected on basis of knowing (or thinking they knew) what it was. Excitedly jumps from one object to another.	Hearing impaired	Some instances occur where facilitators cannot hear. Must always be attentive to patient and ask to repeat if comment not heard (do no just agree).
Correcting statement(s)	Must do this if a fact is incorrect. While patients like to guess they are not always right. Do it in a “good guess but not quite right” way.	Triggering associations	Patients may suddenly remember an event, object, person from their past or something in everyday life. It may be triggered by sight, touch, hearing or smell. This code has links with remembering/reminiscing and making observations.
Acknowledging patient	This refers to the instances where the facilitator does not want to break the flow of info/knowledge coming from patient so simply acknowledges engagement in conversation “yes”, “mm”, “uh-huh” type comments.	Sharing knowledge	This is different to giving information because it is not about facts but rather about the patients’ personal understanding/interpretation of an object or a fact. They often talk about it from first-hand experience or can give an example; they feel comfortable in their own knowledge of it.
Disclosing feelings	The facilitator may disclose their feelings about objects but also about why they chose what they chose and why they omitted objects from the study. For example, facilitators thought medical objects would be inappropriate given the settings but many commented on wanting to see those collections.	Disclosing feelings	The patient could feel disgust at looking at an object, at finding out what it is. Equally, they may disclose any number of feelings; fear, happiness, shock, amazement, etc. The object has obviously engaged with their emotions/feelings and they disclose personal connections or inner most interpretations.
Introducing session/objects	The facilitator takes the opportunity at the start of the session to explain what will happen. They will also tell the patient, when a new object is selected, either by the facilitator or the patient, facts and figures about the object or begin by posing a question “what do you think it is?”	Guessing game	Some patients are keen to guess what the objects are and so it becomes almost competitive (perhaps this is reflexive of their personality?). This code is connected with “being correct”, “seeking validation” and “confirming patient thinking”.
Making observations	The facilitator may make observations about the object as a way of questioning the patient, for example “you can see the wear and tear on it, can't you?” He/she may also make observations about the patient … as a form of questioning.	Remembering/reminiscing	This links strongly with “triggering associations” (RETHINK). Remembering or reminiscing about things is often as a result of an object, or less directly, from a conversation induced by an object. Patients remember facts, events and most often very personal stories.
Referring to aides	The facilitator is not expected to be an expert in all things or in the objects for handling so they may refer to aides like information sheets. This project also used images of things when alive/in use to make observation/engagement/comprehension easier.	Making observations	By handing objects to patients and giving them time to look at each one, patients begin to make observations. This may be about their features, for example, weight, colour, patterns, dimensions. May also relate to other similar objects.
Demonstrating object use	The facilitator may take the object and illustrate how it could be use, which way up it would be, etc. This has links to referring to aides as sometimes objects are compared with photos or similar contemporary objects.	Selecting objects	Patient cannot make up mind/does not seem interested/ bothered, cannot see to select. Some patients ask or are invited to touch/handle objects again. This reaffirms their thoughts/feelings/understanding/curiosity.
		Distracting from session	There are numerous distractions within the settings of the study – all were in hospitals. Other patients, visitors, staff, illness, tiredness, music, lack of enthusiasm.
		Stopping due to illness	Coughing, etc. interferes with session – not quite distraction
		Worrying about handling	Some of the objects may be perceived as fragile and not fit for handling by the patient; some will not touch because they don't like the look of it. However, all objects have been selected for the purpose and the facilitator works to encourage touch and dispel any fears.
		Communicating opinions	This is different to sharing knowledge where the patient talks about something he/she has knowledge about. Here they give their opinion for example “you've got to do it! You never know”. The sentence will often have a ‘because’ in it as they explain their opinion. They also tend to start with “I think” or “I don't think”.

It was hypothesized that for the quantitative analysis, participants would show improvements in psychological well-being and happiness between pre- and post-session measures. The qualitative analysis investigated the processes believed to account for these changes.

### Procedure

The research used a standardized protocol (Appendix 2) developed in other research into heritage-in-health interventions (e.g. Chatterjee & Noble, 2009), with a semi-structured interview format to examine the enrichment potential of museum object engagement. Interview questions were linked to the physical and emotional properties of the objects. Sessions lasting between 30 and 40 min took place during afternoon visiting hours for patients without visitors. Sessions were conducted by female facilitators, one a psychologist, the other a museum professional, engaged as researchers on the project. Both facilitators obtained UK Criminal Records Bureau clearance for working with vulnerable adults and were appropriately trained to undertake the work in a hospital environment, e.g. infection control procedures. The study was approved by the hospital Medical Ethics Committee (Ethics Committee approval MREC 06/Q0505/78) and the study was performed in accordance with the ethical standards laid down in the 1964 Declaration of Helsinki and its later amendments.

## Results

### Quantitative Analysis

Two sets of multivariate analysis of variance (MANOVA) were conducted in SPSS: analysis (i) compared pre- and post-session measures for pooled patient groups (one-way, repeated measures analysis); analysis (ii) compared pre- and post-session measures for separate patient groups (two-way, mixed analysis). Dependent variables were pre- and post-session PANAS positive and negative adjective scores and VAS wellness and happiness scores; each of these measures was analysed separately. Means and standard deviations (SDs) (Table 2) were used to estimate effect sizes (Table 3) based on dividing the mean pre- and post-session differences by pooled SD. Effect sizes (Cohen, 1988), were medium to large for PANAS positive scores and mainly medium to small for the other measures.

**Table 2.** Mean scores (SDs).

	PANAS				VAS			
	Positive		Negative		Wellness		Happiness	
	Pre-session mean (SD)	Post-session mean (SD)	Pre-session mean (SD)	Post-session mean (SD)	Pre-session mean (SD)	Post-session mean (SD)	Pre-session mean (SD)	Post-session mean (SD)
Pooled patient groups	28.06 (7.26)	33.57 (8.73)	14.02 (4.90)	12.43 (4.25)	58.76 (21.61)	64.58 (22.27)	62.05 (21.91)	66.92 (22.15)
Acute and elderly care	26.55 (9.72)	33.91 (9.31)	15.64 (6.41)	13.73 (4.78)	62.73 (26.87)	68.64 (24.91)	62.73 (26.11)	69.55 (23.92)
General oncology	29.63 (7.87)	35.94 (10.95)	12.00 (3.12)	10.50 (1.10)	58.50 (23.12)	64.25 (26.38)	62.00 (23.42)	68.00 (26.89)
Gynaecological oncology	26.87 (7.86)	31.50 (9.20)	14.38 (4.21)	12.62 (3.98)	54.44 (21.77)	62.25 (19.82)	57.25 (21.71)	63.50 (20.88)
Neurological rehabilitation	28.81 (2.07)	32.95 (3.60)	14.65 (5.71)	13.41 (5.78)	60.88 (15.85)	64.82 (19.54)	67.09 (17.61)	67.56 (17.72)

**Table 3.** Effect size estimates on pre- and post-session differences.

	PANAS		VAS	
	Positive	Negative	Wellness	Happiness
Pooled patient groups	0.69	0.38	0.27	0.22
Acute and elderly care	0.77	0.34	0.23	0.27
General oncology	0.67	0.71	0.23	0.24
Gynaecological oncology	0.54	0.43	0.38	0.29
Neurological rehabilitation	1.46	0.22	0.21	0.03

0.8 = large effect size; 0.5 = medium effect size; 0.2 = small effect size (Cohen, 1988).

VAS scores are considered ratio data suitable for parametric testing because assessment is made from zero to 100. PANAS scores use five-point Likert scales normally regarded as ordinal data so homogeneity of variance was checked prior to undertaking parametric tests, given the unequal sample sizes. An *F*-test showed that 2 out of 20 differences were non-significant, implying homogeneity of variance, and as parametric tests are considered robust to minor violations (Howell, 1987), MANOVAs were performed on the data.

*Analysis (i) Pooled patient groups*. Highly significant improvements in all PANAS and VAS measures for pre- and post-session scores comparisons.
*Analysis (ii) Separate patient groups*. No significant differences between patient groups or interaction of patient groups with other variables (Table 4). Most patient groups demonstrated similar levels of improvement on all measures despite some starting from lower baselines, with the exception of neurological rehabilitation participants who showed less improvement on measures of wellness and happiness.

**Table 4.** Significance levels of analyses (i) and (ii).

	PANAS		VAS	
	Positive F value (df) significance	Negative F value (df) significance	Wellness F value (df) significance	Happiness F value (df) significance
(i) Pooled patient groups	69.72 (1,53)	24.82 (1,53)	18.20 (1,53)	10.30 (1,53)
	*p* < .001**	*p* < .001**	*p* < .001**	*p* < .002**
(ii) Separate patient groups	0.60 (3,53)	1.69 (3,53)	0.26 (3,53)	0.30 (3,53)
	*p* < .614	*p* < .181	*p* < .852	*p* < .829

### Qualitative Analysis

The content analysis sought to determine which words were used with the highest frequency to express emotion during the object handling sessions, in particular whether participants used adjectives from the PANAS word lists, alternate forms of these words or synonyms (Table 5).

**Table 5.** PANAS adjectives, alternate words and synonyms.

	PANAS adjectives	Alternate words	Synonyms
Positive words	Active	Activation, activity	Energetic, dynamic, full of life, lively, on the go, perky, up and about, vigorous, with vitality
	Alert	Alerted, alertness	Aroused, awakened, aware, observant, prepared, ready to act, vigilant, watchful
	Attentive	Attend, attending, attention	Concentrating, noticing, observant, focused, listening, thinking
	Determined	Determination	Firm, resolute, indomitable, single-minded, strong-minded, unwavering
	Enthusiastic	Enthuse, enthused, enthusiasm	Animated, eager, keen, fervent, passionate, wholehearted
	Excited	Excite, excitement, exciting	Electrified, energized, motivated, thrilled
	Inspired	Inspirational, inspire, inspiring	Astounded, dazzled, educated, encouraged, informed, motivated, stimulated, stirred
	Interested	Interest, interesting	Absorbed, attracted, captivated, engaged, fascinated
	Proud	Pride, prideful	Confident, delighted, fulfilled, gratified, independent, honoured, pleased, satisfied
	Strong	Strength, stronger, strongest	Passionate, powerful, resilient, resistant, robust, solid, sturdy, tough
Negative words	Afraid	–	Fearful, frightened, petrified, terrified
	Ashamed	Shame, shamed, shameful	Apologetic, embarrassed, humbled, humiliated, mortified, sorry
	Distressed	Distraught, distress, distressing	Anguished, bothered, disturbed, suffering, tormented, troubled, uncomfortable
	Guilty	Guilt, guilt-ridden	Accountable, at fault, blameworthy, culpable, in the wrong, remorseful, responsible
	Hostile	Hostility	Aggressive, antagonistic, argumentative, harsh, intimidating, unfriendly, unreceptive
	Irritable	Irritated, irritability	Annoyed, bad-tempered, cross, petulant, prickly, short-tempered, tetchy, touchy
	Jittery	Jitters	Edgy, fidgety, frazzled, fraught, jumpy, on edge, stressed out, strung-up, wound-up
	Nervous	Nerves, nervy	Anxious, bundle of nerves, concerned, panicky, tense, uneasy, worried
	Scared	Scare, scary, scarify	Alarmed, chilled, daunted, intimidated, jolted, shocked, startled
	Upset	Upsetting	Disappointed, dismayed, grieved, hurt, in a state, offended, sad, tearful, unhappy

Findings showed that participants tended not to mention PANAS adjectives directly but instead used related words to account for their feelings. The word “interested” occurred with the highest frequency, but rather than use the word in this form, participants tended to say “I find this interesting” or “I am fascinated by this”. Overall, however, PANAS adjectives and related words had a relatively low frequency of occurrence within participant discourse (Table 6).

**Table 6.** Frequency of occurrence of PANAS adjectives.

	PANAS adjective	No. participants who used word	No. times word occurred overall
Positive words	Active	1	1
	Alert	1	1
	Attentive	0	0
	Determined	0	0
	Enthusiastic	2	2
	Excited	3	3
	Inspired	0	0
	Interested	14	64
	Proud	1	1
	Strong	0	0
Negative words	Afraid	1	1
	Ashamed	1	1
	Distressed	2	2
	Guilty	1	1
	Hostile	0	0
	Irritable	0	0
	Jittery	0	0
	Nervous	1	1
	Scared	1	1
	Upset	0	0

A search of additional words and phrases from health and well-being literature (Brazier et al., 1992; Kobau, Sniezek, Zack, Lucas & Burns, 2010; NEF, 2009; Watson & Clark, 1999) was carried out in NVivo (Table 7) to further scrutinize the discourse for adjectives used spontaneously by the participants. Of these, three positive words (“amazed”, “happy” and “purposeful”) and two negative words (“painful” and “tired”) were found to occur with the greatest frequency among interactions, with “amazed” being used by the greatest number of participants, although not to the same extent as “interested”.

**Table 7.** Additional adjectives from health and well-being literature.

Source	Additional adjective	No. participants who used word	No. times word occurred overall
Brazier et al. (1992)	Apprehensive	0	0
	Impaired	0	0
	Painful	2	2
Kobau et al. (2010)	Calm	0	0
	Cheerful	0	0
	Peaceful	0	0
NEF (2009)	Autonomous	0	0
	Belonging	0	0
	Competent	0	0
	Meaningful	0	0
	Optimistic	0	0
	Purposeful	1	1
	Trusting	0	0
Watson and Clark (1999)	Amazed	6	17
	Happy	1	1
	Tired	2	2

**Figure 1.**
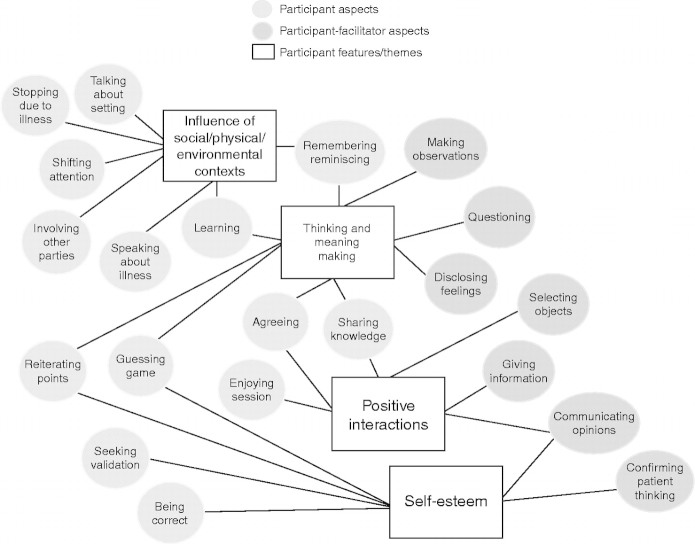
Participant Aspects and Features/Themes.

Twenty-seven codes labelled aspects of the object handling sessions emerged from thematic analysis of the data. Conceptual grouping of these codes produced seven overarching features or themes of the interactive process (Figure 1). Four clear features emerged specific to participants with a further three emerging to explain the facilitator role. Participant features consisted of the “influence of social/physical/environmental contexts”, “thinking and meaning-making”, “positive interactions” and “self-esteem”, whereas facilitator features comprised “encouraging engagement”, “communicating knowledge and information” and “building trust and developing rapport”.

While features appeared distinct and specific to participant or facilitator, interactional aspects of the sessions strongly implied that the features were interlinked. For instance, “questioning” occurred as a feature of “thinking and meaning-making” by the participant, but it also applied to the facilitator role, where questioning techniques were used to encourage engagement and build trust and rapport with participants. Each feature contributed to the process of a handling session, but the balance of features and their frequency of occurrence appeared to affect the outcome. For example, the “communicating opinions” aspect of participant interaction occurred in each of the 16 sessions and 321 times overall. Reporting the qualitative results of the study in their entirety would be lengthy and beyond the remit of a single journal article. Instead, the article focuses on participant themes and highlights important features of the participants’ role in the sessions.

### Thinking and Meaning-Making

Using the above criteria, “thinking and meaning-making” emerged as the most important feature of the patient role in the handling sessions. From the initial codes generated by the qualitative analytic approach, both overt and subtle properties of thinking and meaning-making began to emerge. Participants were able to reminiscence about events, places and people. In this instance, handling a flint hand axe that the participant (PT) discovered was from Sweden, triggered the recall of a visit there.

PT: My husband is a teacher and he spent some time with a group of Swedish teachers as sort of an exchange. One of them had invited us over. We became very good friends … Not for long, it was only four or five days that we went there. We didn't see much but what I did see, it was all very fresh and clean. A very clean place. Very expensive too.

Arguably, remembering this visit had little to do with the flint axe, apart from the fact that it was from Sweden; however, using prior knowledge and acquired experience, the participant developed an interpretation for the object connected with a visit to friends in another country. Remembering events of this sort allowed participants to feel sufficiently comfortable to communicate opinions such as the cleanliness of the country and the cost of living there.

Fifteen of the 16 participants discussed memories or reminisced about life experiences and events while handling the objects. These included childhood memories of days at the beach, learning experiences during school years, artistic tendencies of family members, working abroad, employment skills and hospital and museum visits. While few participants made explicit mention of “learning”, some made connections to learning from the objects and/or the facilitator (FT). In the following extract, an inpatient on a neurological rehabilitation ward is finding out about a fossil vertebra of a marine reptile called an “ichthyosaur”.

PT: Triassic. What does the “assic” stand for then?

FT: Oh yeah, I don't know actually. I don't know what the sic stands for. I know that the Jurassic, that's named after the region where lots of these rocks are from, the Jura in France. I don't know … I don't think Tri is a region like Jura is. I think Tri might be something to do with maybe there's three layers of it or three eras of the Triassic. And then I have no idea about the “sic” bit.

PT: Jura is a place in France?

FT: Yes.

PT: I didn't know that.

Much of the conversation generated in each handling session took the form of learning, whether it was constructing new meanings for objects, sharing facts and ideas or agreeing with the other person's opinions. Learning, meaning-making and thinking tended to be confirmed by the reiteration of points. Participants were seemingly reiterating points to gain assurance from the facilitator that their ideas, opinions and knowledge were correct and that they had understood the information imparted to them. Linked with meaning-making, thought and the reiteration of points is the aspect which describes the process of object identification as a “guessing game”. The guessing game aspect, apparent in all 16 sessions, was often provoked by open questioning from the facilitator such as “What do you think it is?” Participants used prior knowledge to guess what the object was. Other skills were used to identify objects and were interrogated through different senses. The aspect “making observations” spoke most obviously to the visual sense when participants interacted with objects. In the following extract, taken from a session with a patient in an oncology ward, thinking and meaning-making was apparent. The participant not only imparted facts gained from observing the object at close range, but also gave opinions and constructed meaning from acquired knowledge; in this case a greater understanding of the basic anatomy of a tooth.

PT: If you break it on there, it's going to come out a yellow colour. Ours isn't. Our tooth, you cut from here, you only going to get yellow in here, but this …

FT: But this has got more colours.

PT: This has got more colours, yeah.

FT: Now if I hold it up to the light, you've made me see this. It changes. It's quite translucent.

PT: Because all the little lines, looks like the blood goes through, because you said this was cut in half right?

FT: Yeah.

PT: If you joined the other part in there, because this root follows all the way down there.

FT: I wonder if they are little blood vessels?

PT: It could be. And this one moves and this one comes up, so must have some sort of connection.

The aspect “making observations” implies visual examination. However, the data illustrated how visual scrutiny of objects was complemented by the sense of touch. In the extract below, the patient keenly observed a Neolithic axe head, noting the human interaction of the maker with the stone. Touching the object allowed the participant to explore the material used to manufacture the object. The participant's enthusiasm for and description of the axe head conveyed amazement and awe. The participant talked about its physical properties, the skill of the person who made it and the techniques involved. The encounter is typical of a patient–object interaction where the opportunity for hands-on object engagement heightened the participant experience and encouraged a sharing of knowledge, ideas and feelings.

PT: Yeah, when touching I think, whoever made the work on it, whoever done it, the way they done it, obviously at first might just be a big stone, and somebody cut it maybe with the sand and water, cut it through, must have an idea in mind he's going to make this shape of axe or whatever. Must have a skill to do these things and the way he's created this thing is amazing because, look here, they just look like, from here you cannot tell if it a shell or stone or what it is. But when you hold in your hand, my initial reaction was it could be marble, but even in a stone, it's different when you can hold it up. You can actually feel what it is, how created, how it been done.

The ability of participants to closely observe and touch museum objects led to another major aspect of thinking, that of questioning. Ranked second in the list of patient codes, questioning gave patients an opportunity to learn more about the objects, to query their observations or find out about how objects were made and used. The posing of questions by patients indicated engagement and a stimulation of curiosity. Questions increased in number if participants were intrigued by an object. An intriguing object was more likely to augment the communication of personal opinions and feelings. This communication tended to occur further into the handling session, as trust and rapport built up between participant and facilitator. There are many examples that illustrated disclosure as all of the participants revealed their feelings and communicated their opinions multiple times over the course of a handling session. Revealing feelings and communicating opinions were two universal aspects that contributed greatly to the feature of thinking and meaning-making.

PT: I was a bit tired and when they told me I wouldn't be going until tomorrow … because you don't sleep, I thought “not another night of not sleeping”. I was a bit tearful before you arrived so I'm quite glad you came to distract me. Thank you very much.

PT: Well everything's if, if, if, if, and I've been here since something like the 15th September. Then I had to go home and now I'm back. Was so ill with pain and stuff and now I'm still a bit ill, they're supposed to be giving me chemotherapy but nothing's happening so who knows, it's like, who knows.

PT: Um … just like the patterns on them as well actually. It's quite restful looking at it and tactile as well because you have the rough side and the smooth side.

The most frequent participant aspect to emerge, however, was agreeing. Agreeing indicated that the participant was focused on the session, listening to the information, concurring with what the facilitator was saying and demonstrating understanding. Agreeing was potentially indicative of patients thinking about an object, although in some cases it was apparent that a participant's desire to satisfy the facilitator led to an expression of agreement regardless of whether they actually agreed or understood about the object. This tendency to behave in a socially desirable manner probably occurred as a result of participants’ previous experience in social situations and the physical and environmental context. Influence of social, physical and environmental context was another feature affecting the participant role in the handling sessions. Although not always obvious, these contexts potentially had an effect on levels of participant involvement, self-esteem and confidence.

PT: I see yes a spiral. And it's got a big brain, related to the squid and octopus … [reading]

PT: Yeah, that's right, 3500 to 300 BC.

PT: Oh yes it does look lava-ish doesn't it?

## Discussion

It was hypothesized that participants would show improvements between pre- and post-session measures of well-being and happiness. The study demonstrated statistically significant, overall enhancement of psychological well-being as determined by the PANAS measures, and subjective well-being and happiness as determined by the VAS measures. Positive PANAS, wellness and happiness VAS scores increased, and negative PANAS scores decreased in line with predictions, although there were no significant differences between the four patient groups. The average increase in positive mood was greater than the average decrease in negative mood supporting the view of Watson et al. (1988) that the two PANAS scales were independent and orthogonal. Generally, participants reported low levels of negative mood pre-session leaving little room for improvement post-session. Effect sizes carried out on pre- and post-session differences showed a range from small to large although were generally small for the VAS measures, indicating that additional participants and equal sample sizes would be needed to increase the statistical power of the study.

Well-being improvements were demonstrated as a result of participants handling and discussing the objects as well as looking at them, exemplifying the added value of a tactile interaction and a trained facilitator. Criticism of therapeutic interventions suggests that it is the social interaction, not the intervention per se, that brings about beneficial results (Simmons, 2006), but current findings run counter to this claim. Thomson et al. (2012) reported a significant difference between sessions where museum objects were handled and sessions where only photographs were used. Assuming the social element was similar in both conditions, the presence of the objects appears to have further enhanced the intervention benefits. This is supported by the present study when the quantitative and qualitative findings are considered holistically. While quantitative data revealed enhanced psychological and subjective well-being, and increased happiness, qualitative analysis demonstrated that sessions afforded opportunities for thinking and meaning-making through touch. Sessions were characterized by participants communicating their opinions, questioning object facts, remembering and reminiscing. Object-based interaction continually stimulated questioning which gave an impetus for thinking and meaning-making. Simmons posited that the simultaneous presentation of different types of sensory information enhanced the enrichment outcomes and it is likely that these results represent a similar process; object handling focused verbal interaction that enhanced well-being outcomes.

If touch enhanced well-being, it implies the presence of a short-term memory (STM) tactile representation additional to the verbal and visual representations proposed by dual coding (Paivio, 1971, 1986) adding weight to triple or multiple coding models. Craik and Lockhart's (1972) levels of processing model is relevant in that tactile qualities such as texture, shape and weight could have enhanced the kinaesthetic experience of sessions leading to deep and elaborate encoding. Spector et al. (2008) suggested that cognitive stimulation therapy (CST) increased cognitive processing and laid down new connections in the brain as a result of encounters with novel stimuli and social interaction. It is possible that museum object handling and discussion centred on the objects brought about a similar level of cognitive processing. Although it was beyond the remit of the current research to ascertain whether neuronal formation occurred; a future study could employ brain scan techniques (e.g. functional MRI) to examine this possibility.

Results from the quantitative and qualitative analysis inferred that object handling sessions contributed positively to individual participant well-being and that a mixed method approach afforded a more nuanced view of the impact of object handling. While the content analysis found that the frequency of occurrence of PANAS words, alternative word forms and synonyms was low within the 16 participant sessions, other words related to health and well-being (“amazed”, “happy”, “tired”, “pain” and “purpose”) taken from relevant literature occurred frequently within the transcripts. The NEF (2009) identified key indicators to measure the well-being of communities at a national level. The qualitative analysis, conducted as part of this research project, uncovered similar indicators at an individual level represented as features in the context of facilitator–participant interactions. For example, NEF explained that for people to experience personal well-being they need to be engaged in activities, take the opportunity to learn new things and feel that their life has meaning and purpose. The research findings presented here (Figure 1) showed that participants were engaged, contributed to meaning-making and interacted positively.

In keeping with constructivist models of adult learning (Falk & Dierking, 2000; Hein, 1999), participants added to their existing knowledge of objects and linked memories, taking the opportunity to question information in order to construct new meanings. In keeping with Brown and Wragg's (1993) work on the acquisition of learning skills, the majority of questions asked by participants were cognitive in that they were keen to gain knowledge. The research showed that objects taken outside the museum space evoked emotion as well as the recall of events, people and places. Mack (2003, p. 8) referred to the “role of archive material in triggering memories” and Phillips (2008, p. 204) found that when used in reminiscence sessions, museum objects, specifically coins and medals, promoted “learning, creative thought, skills development and greater confidence”. Reminiscence through objects enabled 15 of the 16 participants in the current study to recall and talk about a wide range of memories. The experience of remembering past events adds support to the notion that museum objects accrue multi-layered identities “ranging from conceptual, through the factual, the functional and the structural, to the actual identity” (Maroevic, 1995, p. 24). Participants contributed to these identities by finding meanings in the objects often attributed to personal experiences. The processes of remembering and reminiscing demonstrated how meaning-making could contribute to the beneficial effect of the session and be used “in positive and constructive ways that help build self-esteem and bolster a sense of identity” (Kavanagh, 2000, p. 118).

These outcomes are in line with other similar studies which show that museum objects function as symbols of identity, relationships and society, and that museum object encounters elicit ideas and meaning-making opportunities (e.g. Ander et al., 2012; Dudley, 2010; Froggett et al., 2011; Lanceley et al., 2012; Newman & McLean, 2006). Some authors have argued that meaning-making is important for adjusting to stressful events, such as bereavement (e.g. Gillies & Neimeyer, 2006) and illness (e.g. Lanceley et al., 2012; see references in Park, 2010). The implication of meaning-making in the healthcare setting is explicated by Park (2010, p. 237) who stated that “meaning-making plays a central role in the coping and adjustment of most people facing major life stressors”. Thus, addressing meaning may be a fruitful approach to clinical interventions aimed at helping people recover from these highly stressful experiences.

Throughout the object handling sessions, there were many examples of participants engaging with objects in a multisensory manner. While meanings are normally developed and built upon through the visual sense in a museum environment, object handling in healthcare settings provided participants with the opportunity to experience museum objects through other senses, specifically touch. Arguably, the chance to interact through visual, auditory and tactile senses in an interesting and engaging way with museum objects triggered recall of long-term memories of events and associated meanings. The outcomes of object handling sessions with the patients selected for this study, those in chronic and acute care settings, were not intended as educational or learning experiences, although the presentation of museum objects and related verbal material was in keeping with learning theories concerned with the integration of information and VAK preferences. Furthermore, some of the elements that emerged from the qualitative analysis pointed towards a “community of learning”. This patient–facilitator “community” was transitory, but exhibited some of the features of learning communities, notably the facilitation of “information exchange, knowledge sharing and knowledge construction through continuous interaction, built on trust and maintained through a shared understanding” (Daniel, McCalla & Schwier, 2007, p. 296).

## Limitations and future work

A key limitation of this study was the fact that it was not longitudinal. In a future longitudinal study of 6 months or over, individual case histories could be examined in depth for sustained effects in coping and resistance to negative life experience. Sample size was also an issue, so a future study should consider a randomized controlled trial with a greater number of participants where the object handling intervention could be compared with care as normal.

## Conclusions

The evaluation of a heritage-in-health intervention conducted across four patient groups in the same hospital suggested that museum object handling sessions produced beneficial and therapeutic effects on patient well-being and happiness. Similar increases in psychological well-being across the three positive emotion scales (positive PANAS; wellness and happiness VAS measures) implied that findings were not an artefact of the study but represented real improvement over the duration of the object handling session, although it could not be ascertained whether these effects were short-term or sustained. Specific features of the current study such as meaning-making and links to previously stored memories could be used as a basis for further analysis of verbal discourse from healthcare interventions. Findings added weight to the need for provision of arts- and heritage-in-health activities for communities of hospitalized adults temporarily or permanently excluded from gallery and museum visits. As a non-pharmacological intervention, the results of these object handling sessions have shown that meaning-making and thinking have the potential to help patients cope and take part in a positive experience during their hospital stay.

## Appendix 1

**Appendix:** Transcript Used to Test Coding Manual.

Codes	Interview transcript
Joking/giving info	PT: Hello Mum, hello Dad! They're both dead by the way! [talking into audio recorder]
	EA: Oh. [Laughs] Well it doesn't communicate with the other world.
Enjoying session	
	PT: It's electrical, it might! [Laughs]
Enjoying session	
	EA: Shall I take this off you? Then you can hold the objects.
Questioning/inviting touch	
	PT: Yes, sure.
Agreeing	
	EA: OK. Right.
	PT: [Coughing]
Stopping due to illness	
	EA: Feel free to take a break whenever you want as well. We'll stop.
Giving info	
	PT: I won't be able to stop myself.
	EA: Right so we have a piece of art … if I show you them all and then you can pick the first one you'd like to look at. This is a piece of art and then we have five other objects. So … that's very good. I didn't know you could do that?
Introducing session/object	
Sharing power/passing over control	PT: You can do lots of stuff with that! It can save your legs.
	EA: OK. So which object do you fancy to have a pick up and look at?
Sharing knowledge	PT: Is that bronze? No it's plastic. Nice colour though.
Questioning/inviting touch/sharing power	
	EA: There we go. Have a feel of that. Actually it is very close to the colour bronze, yeah.
Questioning/making observations/disclosing feelings	
	PT: Uh-huh.
Inviting touch/confirming point/thinking/giving info	
	EA: But it's natural. It's a natural thing.
Agreeing/one word answer	
	PT: It's a shield is it?
Giving info	
	EA: Yeah of sorts. A turtle.
Guessing game	
Confirming point/thinking/giving info	PT: From a beetle?
Questioning/guessing game	EA: A turtle.
Correcting statements	PT: A turtle shell? Snapper? A little snapper?
Questioning/guessing game	EA: I guess so … well a hawksbill turtle it's called. It's a very … it's a baby one.
Confirming point/thinking/giving info	PT: Yes.
Agreeing/one word answer	EA: Yes, not got too old. In fact, when it gets bigger it becomes, um, it's the tortoiseshell that you see. You know tortoiseshell, when they get bigger those shells turn into tortoiseshell.
Giving info	
	PT: We kill 'em to make things out of 'em.
	EA: Yes. Are you interested in animals at all?
Sharing knowledge	
	PT: Yeah.
Confirming point/thinking/questioning	
	EA: I think it must be amazing to see turtles in the sea. I've never seen one myself.
Agreeing/one word answer	
	PT: Having been a building engineer and a hobbyist all my life, I wouldn't like to see it like this. I would like to see something made with it.
Disclosing feelings/giving info	
	EA: I see.
Giving info/communicating opinions	PT: Something done with it.
	EA: Oh, yeah. More artefact?
Acknowledging point	
Communicating opinions	PT: I think it should live on. Not as what it was but to progress by someone improving it in a way.
Confirming point/thinking/questioning	EA: Yeah. Make a craft out of it … craft it. Well this comes from the zoology museum so I guess they keep it as a …
Disclosing feelings/communicating opinions	PT: It's nice. I like it.
Confirming point/thinking/giving info	EA: … specimen of …
Disclosing feelings	PT: Of course, yeah.
Giving info	EA: That's interesting though what you're saying. Have you ever made anything out of tortoiseshell?
Agreeing	PT: All the time.
Questioning	EA: Oh really?
	PT: Not necessarily tortoiseshell, no but out of many things.
Giving info	
	EA: Oh. What sort of things do you make?
Questioning	
	PT: Anything.
Giving info	
	EA: Anything that takes your mind off …
Questioning	
	PT: Yeah, not treen but maybe a face or something.
Giving info	
	EA: Oh right. Very creative.
Questioning	
	PT: What else have we got?
Giving info	
	EA: Let's have a look.
Acknowledging point	
	PT: Can you take them out for me? I don't want to …
Selecting object/questioning	
Selecting object/sharing power	EA: It's not just you. They are quite difficult to get out actually. There we go. That's my mystery object I say to everyone.
Questioning/worrying about handling	PT: It's a mystery object is it?
Giving info/inviting touch	EA: Well it's quite difficult for people to guess what it is. Maybe you'll get it? I don't know.
	PT: It's weighty.
Questioning	
	EA: It is.
Giving info/questioning	
	PT: Not too heavy so it's not a meteorite. Well it could be but … it probably isn't heavy enough.
Making observations	EA: It's quite old.
Confirming point/thinking	PT: Yes a lovely shark tooth.
Making observations/guessing game	EA: Shark tooth, you got it! How do you know that then?
Giving info	PT: I don't know.
Agreeing/guessing game	EA: [Laughs].
Confirming point/thinking/questioning	PT: Not a Megaladon is it?
	EA: Yeah. Are you reading this?
Enjoying session	
Seeking validation	PT: I'm not reading. How can I be reading?
Confirming point/thinking/questioning	EA: Because my sheet says sharks tooth, Megaladon with all the …
Giving info/questioning	PT: Well I can't see that.
Giving info/referring to aides	EA: Wow. Well so you know quite a lot about these things then? Or …
Giving info	PT: Let's go! It's your game I'm playing.
Questioning	EA: Yeah [laughs].
Guessing game	PT: Lucky guess.
Confirming point/thinking/enjoying session	EA: Lucky guess. Well I suppose it does look like a tooth. Once you realise it's a tooth and take a guess.
Being correct	PT: Well it took a bit … it's quite heavy. That surprised me.
Confirming point/thinking	
	EA: Yes. Well it's fossilised so it's …
Making observations/disclosing feelings	PT: Absolutely.
Giving info	EA: But um … it's quite a spooky one in some ways, I think. To imagine the size of the whole animal
Agreeing	PT: Would be … you didn't expect me to get that did you?
Disclosing feelings/giving info	EA: No. Some people have got tooth but they've not got shark. Are you into your archaeology and …
	PT: Not unduly. I'm just old.
Guessing game/being correct	
	EA: [Laughs] You're interested in everything.
Confirming point/thinking/being correct/questioning	
	PT: Well you get to learn a little bit of everything I suppose.
Giving info	
	EA: Yeah. Well they've got a really good natural history museum at the university just down the road, the Grant Museum. It's kind of like a mini natural history museum. It's got lots of bits. Right anything else.
Questioning	
	PT: What else have we got?
Communicating opinions	
	EA: You're going for that one?
Giving info	
	PT: Yeah, I'll let you do it.
Sharing power/passing over control	
	EA: There we go. Yeah so that's …
Selecting objects	PT: A little bronze.
Selecting objects	EA: Yeah.
Agreeing	PT: From the bronze age is it? Don't tell me that's a tennis bat he's got in his hand!
Inviting touch	EA: [Laughs] No. They call it a rattle, a religious rattle, because it's a figure of a goddess.
Making observations	PT: Sure.
Confirming point/thinking	EA: Well let's think . . is it bronze age?
Seeking validation/questioning	PT: Well it's bronze.
	EA: It's made of bronze so . . it's 600 bc so that's a bit later than our Bronze Age but it's not from England. I don't know if you can guess where it's from?
Correcting statements/giving info	
	PT: Egyptian?
Agreeing	EA: Yeah, yeah spot on. They did like cats and they had lots of depictions of cats.
Questioning	PT: That's right. I was going there or Inca-ish.
Making observations	EA: Somebody else said that actually and once they'd pointed it out I thought, yeah it does look South American or something doesn't it?
Giving info	PT: Yeah.
Guessing game	EA: But yes, that's a nice Egyptian figurine. It's the sort of thing you would have had in your house to worship …
Seeking validation	PT: A talisman.
Confirming point/thinking/giving info	EA: Yes, exactly.
Being correct/giving info	PT: We're doing well aren't we?
Giving info/questioning	EA: Do you know what the name of the goddess is?
	PT: Oh dear.
Agreeing/one word answer	
	EA: It's not meant to be a test! [laughs]
Giving info	
	PT: Um … no.
Sharing knowledge	EA: This one's called Bastet.
Confirming point/thinking	PT: Baster?
Guessing game	EA: Bastet.
Questioning	PT: Bastet. It's a female bastard?
	EA: Well! But she's goddess of family, birth, lots of other things.
Guessing game	PT: Birth I was going to say birth because of the carrying of the infant. Yes, that makes sense doesn't it?
	EA: Yes she's often associated with kittens or babies.
Giving info	
	PT: Yep.
Hearing impaired	
	EA: We couldn't quite fit a mummy in a box but we managed to get that one in!
Giving info	
	PT: What is that? It looks like a handkerchief. It's not. It's a bit of …
Questioning/joking	
	EA: This is from the geology collection so it's a rock sample. It's quite a pretty one.
Giving info.	
	PT: A bit of mineral?
Making observations/communicating opinions/seeking validation	
	EA: Yes. Have that. See what it feels like.
Confirming point/thinking	
Agreeing/one word answer	PT: It's a petrified mineral or something?
Joking	EA: Yes it is. It's a hardened crystal called agate.
Questioning/making observations	PT: Yes.
Giving info	EA: It's made of silicon and quartz so it's close to other minerals like diamonds I suppose?
	PT: Petrified yeah?
Questioning	
	EA: Yes. It's been polished up a bit so you can see one side's shinier than the other.
Confirming point/thinking/inviting touch	
	PT: Yes.
Seeking validation	
	EA: It comes in lots of different colours. You may have seen it in different colours because it's not the white that makes it agate, it's the banding around here.
Confirming point/thinking/giving info	
	PT: Sure. Absolutely.
Agreeing/one word answer	
	EA: It's distinctive. Have you ever made anything out of stone or mineral?
Giving info	
	PT: Only ashtrays.
Seeking validation	EA: Ashtrays? Oh yeah. Well this could work as an ashtray if you had the other part.
Confirming point/thinking/making observations	PT: Or pot pourri pots. Things like that.
Agreeing/one word answer	EA: So you cut them and polish them?
Giving info	PT: Yeah. Not anymore but I have done.
	EA: Um, interesting. I've always thought that would be a nice …
Agreeing	
	PT: I can't help myself. I'll be on holiday and I'll pick something up or I'll see something and I've just got to open it.
Giving info/questioning	
	EA: Yes well that's how you find these things so yeah. I've never dared do that. I don't think I would be strong enough!
Giving info	PT: You've got to do it! You never know.
Questioning/disclosing feelings	EA: Yeah. You don't know what's in there.
	PT: You'd never have found the first dinosaur leg if you didn't dig up that hole or …
Giving info	
	EA: That's true.
Questioning	
	PT: So which is your favourite piece in here? You haven't shown me that one yet.
Agreeing/remembering/reminiscing	
	EA: Oh yeah, that's a nice one. I quite like that one actually.
Communicating opinions	
	PT: Axe head from the Stone Age?
Remembering/reminiscing	
	EA: Well actually it is a bit later.
Confirming point/thinking/disclosing feelings	PT: A bit later than Stone Age are we?
Communicating opinions	EA: Yeah, it's Iron Age but obviously they were still using stone.
Confirming point/thinking	
	PT: Flint?
Communicating opinions	
	EA: Yeah. I mean they have iron but this is made of stone obviously.
Confirming point/thinking	
	PT: A trick question! A very nice one. Well worked.
Questioning/selecting objects	
	EA: Um.
Confirming point/thinking/disclosing feelings	PT: Been used.
Seeking validation	EA: Yep.
Correcting statements	PT: So it wasn't just …
Questioning	EA: It was obviously sharp at the beginning but that … you can see the wear and tear on it can't you?
Confirming point/thinking/giving info	PT: Yeah.
Seeking validation	EA: And I expect you can feel that polish on it?
Confirming point/thinking/giving info	PT: Oh yeah. And the hammer end. Wherever it was shafted there will be a smoother part where it was bounded with the leather or something.
Guessing game	EA: Oh yes.
Acknowledging point	PT: There.
Making observations	EA: Do you think … that would be the bit there?
Confirming point/thinking	PT: Yes.
Questioning	EA: Yes. Oh yes.
Giving info/making observations/questioning	PT: Wouldn't it?
Agreeing/one word answer	EA: Yes, you're the first person to point that out actually. Well the first picture …
Inviting touch	PT: Oh you've got a picture of it? You're keeping them from me aren't you!?
Agreeing/sharing knowledge	EA: I keep forgetting that they're there. You can see that would have been the shaft bit and two pieces of wood up there.
	PT: So it would have been shafted there and it would have been bound on?
Confirming point/thinking	EA: Yeah and that would have worn it away I suppose.
Making observations	
	PT: Yes, it would be smooth wouldn't it?
Questioning	
	EA: Yes.
Agreeing/one word answer	
	PT: Yeah, this blade occurred more recently. I could see that because it … the chip wouldn't have been so sharp if was done a long time ago.
Confirming point/thinking	
	EA: Oh I see, it would have worn away?
Seeking validation	
	PT: Yeah it would have been smoother, the chip. Somebody's dropped it.
Confirming point/thinking/giving info	
	EA: Yeah, you're like Sherlock Holmes actually. Picking up lots of these things really just by looking at them. I think that probably comes from working things yourself doesn't it? You can see the materials you work with. How interesting. Do you like archaeology?
Referring to aides/questioning/joking	
	PT: Well I told you, I'm just old.
Giving info/referring to aides	
	EA: [Laughs].
Questioning/making observations	PT: So I know nothing. I might be a plant.
	EA: [Laughs] Oh we still haven't done the art. Do you want to have a look at that?
Confirming point/thinking/giving info	
	PT: The art?
Agreeing/seeking validation	
	EA: It's the last one.
Confirming point	
	PT: Yeah, what have you got? A rubbing?
Making observations/communicating opinions/sharing knowledge	
	EA: Well it's not a rubbing. It's a print.
Questioning	
	PT: A print. That looked like a rubbing under the paper.
Sharing knowledge/making observations	
	EA: Yeah absolutely. So that's the print that came from this plate. This is the plate.
Confirming point/thinking/making observations/	
	PT: The plate's actually survived.
Questioning	
	EA: Yes.
Disclosing feelings	
	PT: I'm not going to stain it?
Enjoying session	
	EA: No.
Communicating opinions	
	PT: So this was etched?
Selecting objects	
Questioning	EA: Yeah, it's an etching.
Questioning	PT: And inked?
Giving info	EA: Yeah.
Agreeing/questioning	PT: Etched and inked.
Correcting statements	EA: So you used the acid … do you know the process?
Questioning/making observations	PT: Basically …
Confirming point/thinking/giving info	EA: Well you cover this with wax to start off with, you dig your design into the wax, then dip it into an acid bath and that actually cuts away into the copper, where you've cut into the wax.
Questioning	PT: Where you've cut into the wax, yeah.
Confirming point/thinking	EA: Then cover it in ink and then wipe off most of the ink and then all the ink stays in the little grooves and then that's what's printed on there. It's quite an intricate process.
Worrying about handling	PT: And you make it one out of fifty.
Confirming point/thinking	EA: Yeah, you have to re-ink it I guess.
Questioning	PT: Re-ink it.
Confirming point/thinking	EA: You can definitely see the differences … the collection is quite interesting. You can see the differences in the early prints and later ones because the ink literally starts to run out and gets lighter and lighter.
Questioning	PT: Absolutely. Oh my. Why would someone go to such great trouble to do that?
Confirming point/thinking	EA: Yeah.
Reiterating point	PT: What is it that they are actually trying to show you behind? Is it the works behind?
Questioning	EA: Yeah. Because he could have just drawn it but it's interesting that you would choose to do …
Agreeing	PT: Conceal it?
Giving info	EA: Yes. Interesting.
	PT: Beautiful work.
Reiterating point	EA: Yes, it's very skilful.
Giving info	PT: Absolutely. The straight lines and the nuts and bolts of the … even the twisting the mill.
	EA: Yeah, gosh I mean to think you're not drawing that, you're … that's in wax.
Questioning	PT: Absolutely. Much harder.
Giving info	EA: Yeah. Well this guy was quite a child star. He joined the Slade School of Art …
Reiterating point	PT: Child star?
Making observations	EA: Well not quite child. He was 16 when he joined the Slade School of Art, which was much younger than most people.
	PT: It's excellent. May I look at it please?
	EA: Yeah, have a look at that one.
Agreeing/questioning	
	PT: Oh yeah.
Confirming point/thinking	EA: Do you recognise the place at all? I ask people that. I don't know whether you've been there?
Questioning	PT: No. I was trying to. I was trying to.
	EA: It's Teddington Lock or Weir.
Confirming point/thinking giving info	
	PT: Teddington Lock is it?
Questioning	
	EA: I don't know if you've ever been? I've never been there actually so I wouldn't recognize it.
Confirming point/thinking	
	PT: I've been to Teddington Lock but I didn't recognize it.
Disclosing feelings	
Confirming point/thinking	EA: It's called Teddington Weir in this.
Agreeing/making observations	PT: The weir is behind.
	EA: Is that different because …
Confirming point/thinking giving info	
	PT: We can see the weir behind can't we?
Agreeing/communicating opinions	
	EA: Oh that bit there?
Confirming point/thinking giving info	
	PT: That would be the weir.
Questioning	
	EA: And this is the lock. That's right.
Giving info	
	PT: Right. Yeah. It's very good isn't it?
Disclosing feelings/questioning	EA: Yes it is isn't it? It's quite nice to see the two together actually because you usually see the end product.
Inviting touch	PT: Absolutely, yeah.
Agreeing	EA: It's quite nice that the art collection have kept the plate as well so. That's the last object so I don't know if you want to hold anything again or are you ready to finish?
Questioning	PT: No, I'm happy. You've made my day.
	EA: Oh good.
Making observations	
	PT: I'd like another look at the sharks tooth actually.
Giving info	
	EA: Yeah, go for it. Yeah.
Questioning	
	PT: Um.
Questioning/giving info	
	EA: Have you ever swum with sharks?
Giving info/remembering/reminiscing	PT: No.
Giving info	EA: A woman the other day had …
Making observations	PT: Away from them! [laughs]
Questioning	EA: [Laughs] Absolutely.
Questioning	PT: I've fished for them but I've never swam with them.
Questioning	EA: Oh really, you've fished for them? Yeah.
Sharing knowledge	PT: I've fished for the smaller ones like spurdog and the tote and things like that but not for the larger ones.
Making observations	EA: No.
Agreeing/disclosing feelings	PT: I wouldn't fish for something I can't eat. I can't eat a shark. Not a whole shark.
Confirming point/thinking/disclosing feelings	EA: Not one for dinner?
Agreeing	PT: I was never one for wanting killing.
Disclosing feelings/giving info	EA: Yeah.
Inviting touch	PT: I wouldn't go and kill a load of fish.
Disclosing feelings/enjoying session	EA: Yeah and then do nothing with them.
	PT: I might catch a couple for dinner.
Re-viewing objects	
	EA: Yeah I think that's quite nice actually.
Giving permission	
	PT: That's good fun.
	EA: Yeah and there's a use for that.
Questioning	
	PT: It's like killing two women when you only need one! [laughs]
One word answer	
Giving info	EA: [Laughs]
Joking	PT: I'm joking! Of course, he's got a never-ending supply of
	these, this chap?
Enjoying session	Ea: Yeah. Well somebody told me that they re-grow. Is that right?
Giving info	PT: Yeah. They just … one breaks and they just grow.
Questioning	EA: Amazing isn't it? I wish we could have that.
Giving info/remembering/reminiscing	PT: Well I've got mine coming next week! [laughs]
	EA: [Laughs]
Communicating opinions/joking	PT: So we have really got that.
	EA: I suppose so. The human brain has thought up a solution to it.
Joking	
Giving info	PT: I've got everything coming. A lot happening nowadays. I'm having new lungs as well. It just shows you don't it?
Confirming point/thinking	EA: Absolutely.
Disclosing feelings	PT: Everything we touch. I'm holding your … I really enjoyed that. Thank you very much.
Confirming point/thinking	EA: Oh good. Something a bit different?
Communicating opinions	PT: It gave me something to do that I never had to do before.
Disclosing feelings	EA: Yeah.
Disclosing feelings	PT: I enjoyed it. It didn't just take up ten minutes. I actually enjoyed the time.
Confirming point/thinking	EA: Oh good. That's what we want to find out as well. We're just … it's just a mad idea, whether it's actually interesting.
Joking	PT: I don't think it's a mad idea at all. Anybody who can … with intellect … a small one, can get into something in
	here. You're going to come across people who go, “I hate that”. You're going to get it. And then you're going to get someone who's going to fall all over it and not going to let you go. You know? EA: Yeah exactly.
Enjoying session	
	PT: But I thoroughly enjoyed it.
Making observations/questioning	
	EA: Oh good. That's really good news.
Giving info/questioning	PT: So how many more boxes have you got?
Agreeing/sharing knowledge	EA: Well we've got a couple.
Disclosing feelings	PT: Oh well I'll see you again.
Giving info	EA: We'll come around again.
Enjoying session	PT: Yeah I'll see you again.
Communicating opinions	EA: My colleague, who's got the other box, she's talking to that gentleman over there. Are we OK to do the … measures again?
Confirming point/thinking/giving info	PT: Yeah. Do they actually pay you to do this?
Giving info	EA: Yeah.
	PT: That's alright then.
Seeking validation	
	EA: They got a grant.
Confirming point/thinking	
	PT: Give us a job. I could do that. Get a grant!
Enjoying session	EA: It's a research grant so it's … we've got to come up with the goods afterwards to say “oh yes it definitely helps people”.
Questioning	PT: I think it does. Especially if you've got somebody a bit down in the dumps and a bit … Somebody might not be getting visitors so something like that.
Giving info	EA: Yeah.
Acknowledging point	PT: Some people don't. I've noticed that they don't get any. It might …
	EA: It's a long time to spend by yourself?
Enjoying session	
Giving info/disclosing feelings	PT: Can be yeah, can be.
Communicating opinions	EA: So it's … right so
Seeking validation	
Confirming point/thinking	
Enjoying session	
Disclosing feelings	
Questioning	
Giving info	
Giving info	
Giving info	
Agreeing/giving info	
Questioning	
Agreeing/questioning	
Confirming point/thinking	
Communicating opinions	
Giving info	
Communicating opinions/joking	
Giving info	
Communicating opinions/	
sharing knowledge	
Acknowledging point	
Sharing knowledge	
Making observations	
Agreeing/communicating	
opinions	

## Appendix 2

Object Handling Protocol.

1. The object handling session begins with a general introduction and explanation of the project. The patient is asked whether they would like to participate and if so is given the patient information leaflet (PIL) that outlines the project in writing. They are asked to read the PIL and whether they have any questions or concerns. The PIL is for the patient to keep. If the patient has further questions these are addressed in a more detailed, step-by-step overview of the session. The patient is asked to read and sign the Consent Form where they agree to being audio recorded and their data being used for research. The recorder is turned on.
2. The facilitator asks the participant to complete the Positive Affect Negative Affect Scale (PANAS) and the Visual Analogue Scales (VAS) for Wellbeing and Happiness.
3. While/after the box is unpacked, the facilitator asks:
  - How do you feel about handling museum objects?
  - Have you handled museum objects before?
  - Do you visit museums?
4. Once the objects are laid out on the mat, the facilitator asks the following questions:
  - Would you like to take a look at the objects and choose one to handle (first)?
  - What does the object feel like?
  - What do you find interesting about it?
  - What do you feel about the object?
  - What attracted you to this object?
  - *What do you think this object is?* Additional questions/prompts to emphasise connections with other people/places/times:
  - Do you have any questions about the object(s)?
  - Can you think of any experience that might relate to this object?
  - Where do you think it comes from?
  - What material do you think it is made out of?
  - What do you know about this material?
  - What use do you think the object would have?
  - Have you seen an object like this before?
  - What does it remind you of?
  - *Do you have any other questions about the object(s)?* The facilitator asks the patient to fill out the PANAS and VAS after which the facilitator asks:
  - Do you think these sessions are a good idea?
  - *Did you enjoy the session?* The audio recorder is turned off (if no audio recorder is used, the facilitator writes up the key outcomes of the session).
